# Characterization of Human Knee and Chin Adipose-Derived Stromal Cells

**DOI:** 10.1155/2015/592090

**Published:** 2015-02-03

**Authors:** Magali Kouidhi, Phi Villageois, Carine M. Mounier, Corinne Ménigot, Yves Rival, David Piwnica, Jérôme Aubert, Bérengère Chignon-Sicard, Christian Dani

**Affiliations:** ^1^iBV, UMR CNRS/INSERM, Faculté de Médecine, Université Nice Sophia Antipolis, 06107 Nice Cedex 2, France; ^2^Research, Galderma, Sophia Antipolis, 06410 Biot, France; ^3^Plastic, Reconstructive and Hand Surgery Department, Saint Roch Hospital, 06000 Nice, France

## Abstract

Animal study findings have revealed that individual fat depots are not functionally equivalent and have different embryonic origins depending on the anatomic location. Mouse bone regeneration studies have also shown that it is essential to match the *Hox* code of transplanted cells and host tissues to achieve correct repair. However, subcutaneous fat depots from any donor site are often used in autologous fat grafting. Our study was thus carried out to determine the embryonic origins of human facial (chin) and limb (knee) fat depots and whether they had similar features and molecular matching patterns. Paired chin and knee fat depots were harvested from 11 subjects and gene expression profiles were determined by DNA microarray analyses. Adipose-derived stromal cells (ASCs) from both sites were isolated and analyzed for their capacity to proliferate, form clones, and differentiate. Chin and knee fat depots expressed a different *HOX* code and could have different embryonic origins. ASCs displayed a different phenotype, with chin-ASCs having the potential to differentiate into brown-like adipocytes, whereas knee-ASCs differentiated into white adipocytes. These results highlighted different features for these two fat sites and indicated that donor site selection might be an important factor to be considered when applying adipose tissue in cell-based therapies.

## 1. Introduction

Autologous fat grafting remains the gold standard therapy for soft tissue defects, correction or, augmentation in reconstructive and plastic surgery. Autologous fat grafting is particularly useful after tumor removal, for breast reconstruction surgery after mastectomy, to repair extensive facial deformities caused by injury, illness, or congenital abnormalities. With some variations, the technique consists of three stages: fat harvesting from any donor site, processing of the aspirate, and reimplantation at a site that differs from the donor site [1]. Enriching fat grafts with adipose-derived-stromal cells (ASCs) dramatically enhances fat graft survival [2]. A commercial device for ASC isolation and reinjection preparation has recently received FDA approval, illustrating the increased use of adipose tissues and ASCs for a broad range of cell-based therapies [3]. The treatment's main advantages are that autologous fat is easy to obtain in large quantities in various parts of the body with minimum morbidity for patients. However, there are still controversies and unresolved questions regarding autologous fat grafting due to the unpredictability of postoperative outcomes. The main disadvantages of this technique are variable engraftment and resorption rates, microcalcification, and cyst formation due to fat necrosis [4]. The differentiation of ASCs toward an undesirable cell type after grafting cannot be ruled out. Indeed, clinical results are unpredictable, without any clue about the reasons underlying the reconstruction success or failure. Greater cellular and molecular knowledge regarding adipose tissues is essential to improve postoperative outcomes.

Two functionally different types of adipose tissue—brown and white—coexist in mammals. Both are involved in the energy balance but have opposite functions. White adipose tissue is mainly involved in energy storage and mobilization in the form of triglycerides. In contrast, brown adipose tissue burns fat and is specialized in energy expenditure. In this key thermogenic organ, brown adipocytes convert nutrients into heat by uncoupling respiration from ATP synthesis. This process is mediated by the brown adipocyte-specific uncoupling protein (UCP)1 and is stimulated through a PKA-dependent pathway [5]. Human brown adipose tissue represents a minor fraction of adipose tissue, is scattered throughout the body, and disappears from most areas with age, persisting only around deeper organs [6]. White adipose tissue is more abundant, dispersed throughout the body, and functional differences between fat depots have been reported. Individual white adipose tissues are not equivalent, in terms of ASC abundance, proliferation, and differentiation [7, 8]. Recent published data, including ours, demonstrated different embryonic origins for adipocytes in rodents [9, 10]. Contrary to the previous belief that adipocytes derive from mesoderm only, we have shown a neuroectoderm origin for cephalic adipocytes [11]. It has been proposed that embryonic origins could play a critical role in differences observed between fat depots.

Despite the heterogeneity in fat depots, it is still considered that any adipose tissue site, such as abdomen, knee, and hip, could be used as a fat depot donor site for facial transplants. The donor site is usually selected by the surgeon based on the quantity of fat needed or on the patient's preference. The question regarding the best donor site for grafting to a heterotopic site remains unclear because no scientific studies have adequately addressed this issue.

Homeobox (*Hox*) genes encode transcription factors determining the positional identity along the anterior-posterior body axis of animal embryos [12], and recent studies revealed that they also have prominent roles in adult cells [13]. The* Hox *code, that is, the* Hox* gene expression profile, has been shown to play a critical role in stem cell positional identity. More importantly, this positional identity is retained after transplantation [13]. Leucht et al. illustrated the critical role of* Hox* code, showing that transplanting tibia-derived* Hox*-positive osteogenic stem cells into a* Hox*-negative site in mandible leads to aberrant bone regeneration [14]. The influence of the* Hox* code has also been highlighted in wound healing [15, 16]. Overall, these animal model studies have highlighted that a* Hox *code mismatch can prevent grafted stem cells from participating in tissue regeneration. The* HOX* code matching between donor and host fats remains to be determined.

Therefore, the cell sources of adipose tissues from different body sites that are used for transplantation at heterotopic sites may not be equivalent. Molecular characterization of tissues from donor and host fat sites that are used for autologous fat transplantation in reconstructive medicine is crucial. In the present study, we compared two human fat sites—limb and face—as a first step to investigate the therapeutic properties of fat according to its anatomical localization.

## 2. Materials and Methods

### 2.1. RNA Preparation

Chin and knee paired fat depots were collected from 11 Caucasians women (age 64 ± 13 years, BMI 21.9 ± 1.5 Kg/m^2^) who underwent elective liposuction procedures, after obtaining their informed consent. Fat tissues were lysed by the addition of  TRIzol reagent (Invitrogen, Life Technologies Corporation, Carlsbad, CA, USA) and RNAs were purified on RNeasy Plus Universal columns (Qiagen, France), according to the manufacturer's instructions. Concentrations of total RNA samples were first evaluated using a Nanodrop spectrophotometer (Thermo Scientific, Waltham, MA, USA) and RNA samples were run in an RNA nanochip in a 2100 Bioanalyzer System (Agilent Technologies, Santa Clara, CA, USA) to verify the RNA sample integrity. All samples displayed good integrity with a measured RNA integrity number (RIN) > 6.

### 2.2. Whole-Genome Transcriptome Analysis

The Affymetrix analysis was performed with 11 paired samples of chin and knee fat tissues.

*Affymetrix Probe Synthesis, Hybridization, Scanning, and Quality Controls*. cRNA labelling was achieved starting from 300–500 ng of total RNA from samples. RNA was converted to double-stranded cDNA with a T7-(dT) primer. cDNA was* in vitro* transcribed to biotinylated complementary RNA (cRNA) by incorporating biotin-CTP and biotin UTP using the Affymetrix IVT labelling kit. 15 *µ*g of biotin-labelled RNA were fragmented to 200 bp size by incubation in fragmentation buffer containing 200 mM Tris-acetate pH 8.2, 500 mM potassium acetate, and 500 mM magnesium acetate for 35 min at 94°C prior to hybridization. For the Affymetrix probe hybridization step, fragmented biotinylated RNA was assessed for relative length on an Agilent 2100 bioanalyzer and hybridized on Affymetrix U133 Plus 2.0 chips for 16 h at 45°C, washed and stained on an Affymetrix fluidic station, and scanned using an Affymetrix genechip scanner. Affymetrix RMA software was used to generate the cell files and to control the data quality prior to the data analysis. These controls included: back_spikes, Housekeeping genes, pm_mean, Mad_residual_mean, Rle_mean, and Box Plot. Validation of all chips was achieved using the principal component analysis (PCA) which is a mathematical procedure based on an orthogonal transformation to convert a set of observations of possibly correlated variables into a set of values of uncorrelated variables called principal components.

*Normalization of the Affymetrix Data.* All 22 chips were normalized with the Array Studio software package (OmicSoft Corporation, USA) using the three-step robust multichip average (RMA) method: background adjustment, quantile normalization, and finally summarization [17].

Only Affymetrix identifiers (IDs) with expression level ≥2^6^ in at least 6 samples in at least one condition were selected for statistical analysis, resulting in data for 29131 out of 54675 IDs present on the HG_U133_Plus_2 chip.

### 2.3. Isolation of Adipose-Stromal Cells and Gene Expression Analysis by Real-Time PCR

Adipose-stromal cells (ASCs) were isolated from 9 paired chin-knee adipose tissues and expanded as previously described [18, 19]. Briefly, fat pads were dissociated with 200 mg/ml collagenase and the stromal vascular fraction (SVF) was separated from the adipocyte fraction by low speed centrifugation. Then, the SVF was seeded onto tissue culture plates and cells were maintained in low DMEM supplemented with 10% fetal bovine serum. After reaching 80% confluence, cells were dissociated, split, and expanded in the presence of 2.5 ng/ml FGF2. This passage was designated passage 1. RNAs were purified on RNesay columns (Qiagen, France). Real-time PCR assays were run on a StepOnePlus system (Applied Biosystems, Life Technologies, France). Transcript expression levels were evaluated using the comparative CT method (2-deltaCT). Delta Ct values were used when “expression relative to* TBP*” is indicated.* TBP* was used for sample normalization.

### 2.4. ASC Proliferation, Clonogenic and Differentiation Assays

Chin- and knee-ASCs were split every 4 days. The estimated population doubling time was 30 hours, and analyses were carried out between passage 2 and passage 4.

For proliferation tests, cells were plated on 24-well pates at 5000 cells/well in proliferation medium (DMEM medium supplemented with 10% FCS). Cell numbers were counted at different times after plating using a particle counter (Coulter Beckman).

For the fibroblastoid colony-forming unit assays, cells were plated at 1000-cell density in a 10 mm diameter culture dish in proliferation culture medium. Ten days later, cells were fixed with phosphate-buffer containing 4% formaldehyde and then stained with crystal violet 0.1% before counting.

For adipocyte differentiation, cells were plated on 24-well plates at 100 000 cells/well in proliferation medium. When cells reached confluence, culture medium was replaced by adipogenic differentiation medium composed of proliferation medium supplemented with dexamethasone (0.25 *µ*M), isobutylmethylxanthine (500 *µ*M), human recombinant insulin (0.34 *µ*M), triiodothyronine (0.2 nM), and rosiglitazone (1 *µ*M). The adipogenic differentiation medium was changed twice a week. After 10 days, cells were fixed with Oil red O [19] or lyzed for RNA preparation.

### 2.5. UCP1 Protein Expression in Differentiated ASCs

ASC-differentiated cells were harvested at day 16 after adipogenic induction. Proteins were extracted and 100 *µ*g protein per condition was separated by 15% SDS-polyacrylamide gel electrophoresis. Proteins were then transferred to polyvinylidene difluoride membrane. The membrane was first incubated with an antibody against UCP1 (Calbiochem) and was then incubated with a secondary antibody rabbit IgG-conjugated horseradish peroxidase (Santa Cruz Biotechnology, Santa Cruz, CA, USA). The membranes were treated with the chemiluminescent HRP substrate (Millipore) according to the manufacturer's instructions. Tubulin was detected as a control with tubulin antibody (Santa Cruz Biotechnology, Santa Cruz, CA, USA).

### 2.6. Triglyceride Accumulation and Lipolysis Assays

ASCs were induced to undergo differentiation as described above. At day 16 after induction, cells were harvested and the intracellular triglyceride concentration was measured using the Triglyceride Colorimetric Assay Kit (Cayman Chemical) according to the manufacturer's instructions. For lipolysis assays, cells were treated with 10 *µ*M forskolin for 4 h and the secreted glycerol concentration was measured in culture supernatant using the Free Glycerol Reagent Kit (Sigma-Aldrich) according to the manufacturer's instructions.

### 2.7. Statistics Analysis

Statistical analysis of the normalized Affymetrix data: for the knee versus chin comparison, a two-sided paired *t*-test was applied using Array Studio software. The Benjamini-Hochberg procedure was used for multiple testing corrections [20]. False discovery rate (FDR) control is a statistical method used in multiple hypotheses testing to correct for multiple comparisons. InStat3 software and a nonparametric unpaired test (Mann-Whitney) were used for statistical analysis of real-time PCR data. *P* values are indicated on each figure.

## 3. Results and Discussion

### 3.1. Large-Scale Affymetrix Transcriptomics Revealed Differential Expression of* Pax3* and* HOX *Genes in Human Chin and Knee Fat Depots

**Figure 1:**
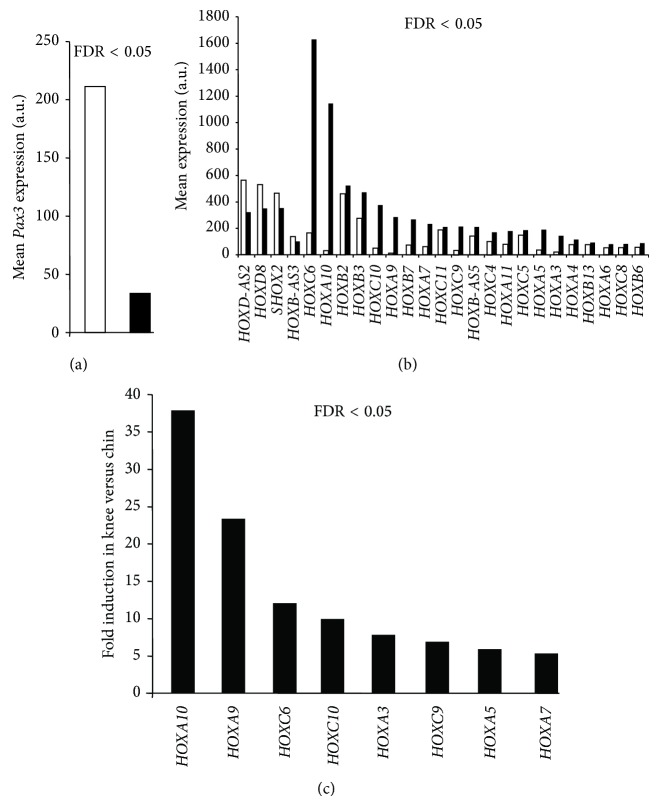
*Pax3* and* HOX* gene expression in paired chin and knee fat depots from large-scale Affymetrix transcriptomics. (a) Mean expression of* Pax3* in chin (open bar) and in knee (black bar) fat depots (*n* = 11 paired). (b) Mean expression of* HOX* genes in chin (open bars) and knee (black bars) (*n* = 11 paired). (c)* HOX* genes with the highest differential expression are presented.

A large-scale transcriptomic analysis was performed using Affymetrix technology to compare the transcriptome from knee versus chin adipose tissues. A total of 346 genes (determined from the 486 modulated Ids) were found to be differentially expressed with a fold < 2 and a FDR value <0.05 (all differentially modulated ids available on demand). These 346 differentially expressed genes were equally distributed between upregulated and downregulated genes (181 up- and 165 downregulated genes). Among the TOP50 upregulated genes were mainly found homeobox genes and inflammatory-related genes. By contrast, most of the genes found in the TOP50 downregulated genes belonged to the “muscle function” (Table 1). Interestingly, microarray analysis revealed that* Pax3* expression was enhanced in chin fat compared to knee fat (Figure 1(a)). Lineage tracing experiments in mice allow establishing a neuroectodermic/neural crest and mesodermic embryonic origins for adipose tissues located in the face and trunk, respectively. As lineage tracing is impossible in humans, only specific marker expression could be used to propose an embryonic origin of human samples.* Pax3 *expression was shown to be associated with neural crest development in humans and to be maintained in adult progenies [21].* Pax3* thus represents a unique neural crest marker in adult humans. Therefore, microarray analysis data strongly suggest that chin fat, but not knee fat, originates from the neural crest. This hypothesis is in agreement with what we observed in mice [11].* HOX* gene expression profiles were then compared in paired adipose tissue samples. Thirty-eight* HOX* genes were detected on Affimetrix chips and 25 of them displayed significant differential expression between both sites (Figure 1(b)). Among those, 4* HOX* genes (*HOXD-AS2*,* HOXD8*,* SHOX2,* and* HOXB-AS3*) were 1.4- to 1.8-fold more expressed in chin fat than in knee fat. In contrast, 21* HOX* genes were preferentially expressed in knee fat depots, with the 8* HOX* genes,* HOXA10*,* HOXA9, HOXC6, HOXC10, HOXA3, HOXC9, HOXA5,* and* HOXA7* being the most differentially expressed (Figure 1(c)). The whole genome transcriptomic analysis therefore revealed that chin and knee fat depots expressed a different* HOX *code. This* HOX* status in human chin and knee fats resembles the* Hox *code identified in mouse mandibular and tibial osteogenic progenitors [14]. It should be noted that the* Hox *code mismatch was responsible for aberrant tibial progenitor differentiation when transplanted into the face.

### 3.2. Differential* Pax3* and* HOX *Gene Expression Was Conserved in Adipose-Stromal Cells Derived from Chin and Knee Fat Depots

**Figure 2:**
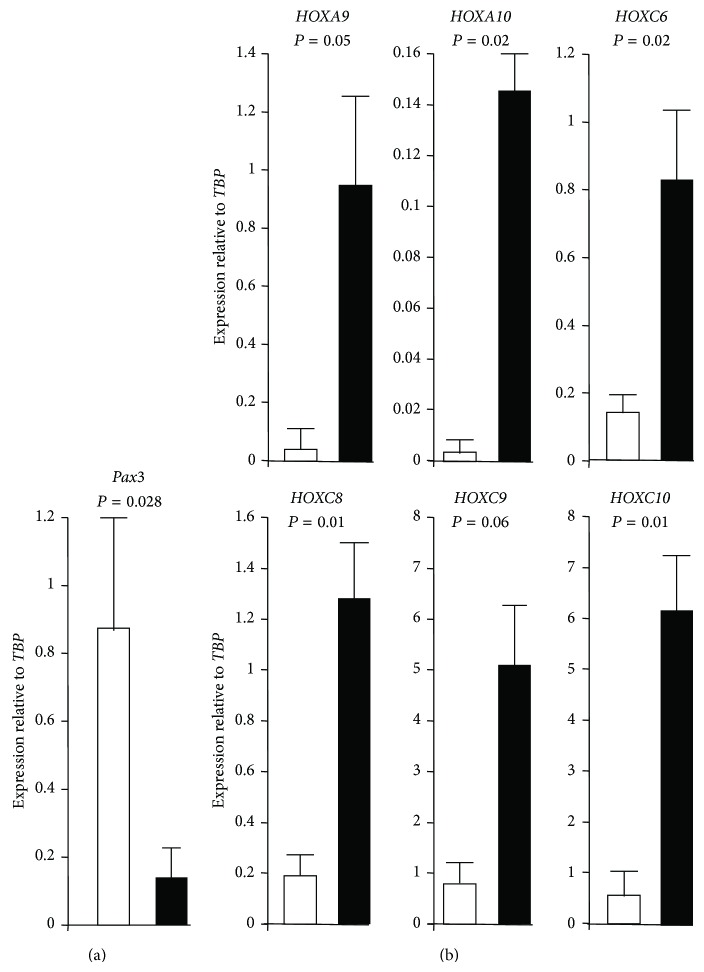
Expression of* Pax3* and* HOX* genes in adipose-stromal cells derived from chin and knee fat: ASCs were isolated from chin (open bars) and knee (black bars) paired fats and were expanded* ex vivo*. (a)* Pax3* expression was quantified by real-time PCR and normalized with* TBP *housekeeping gene. Values are means ± SEM. *P* is indicated (*n* = 4 paired depots). (b) Expression of indicated* HOX* genes was quantified by real-time PCR and normalized with TBP housekeeping gene. Values are means ± SEM. *P* are indicated (*n* = 3 paired depots).

As adipose tissues contain several cell types, such as adipose-stromal cells (ASCs), adipocytes, endothelial cells, and immune cells, we wondered whether chin and knee molecular signatures revealed by microarray data were conserved in ASCs and derived adipocytes. For that purpose ASCs were isolated from the stromal vascular fraction of paired chin and knee fat samples. The skeletal muscle signature of chin fat depot was not detectable neither in undifferentiated ASCs nor in their differentiated progenies (not shown), indicating that other cell types in chin fat biopsies could be responsible for muscle gene expression. In contrast,* Pax3* and* HOX* differential expression was conserved in ASCs.* Pax*3 expression was analysed by real-time PCR and its gene expression was normalized according to* TATA box binding protein* (*TBP*) gene expression, that is, a housekeeping gene used as reference gene. As shown in Figure 2(a),* Pax3* was expressed significantly higher in chin ASCs than in knee ASCs (6.2-fold), supporting the neuroectoderm origin of chin-ASCs.

It has been previously reported that fat from different depots displays distinct developmental gene expression profiles, including* Hox* genes [10, 22, 23], but limb and facial fat were not included in these analyses. We performed real-time PCR to monitor the expression of 6* HOX *genes that displayed the highest differential expression between knee and chin according to the microarray data. As shown in Figure 2(b),* HOX *genes were expressed at different levels. More importantly,* HOX* gene expression was significantly enhanced in knee ASCs compared to chin ASCs (more than 20-fold for* HOXA9 *and* HOXA10 *and between 5- and 10-fold for* HOXC6, HOXC8, HOXC,* and* HOXC10*). These data are in line with the* HOX *expression revealed by the Affimetrix analysis from whole fat depots (compare Figures 1(c) and 2(b)), indicating that the* HOX* code of the whole fat depot reflected the* HOX* code of ASCs. The* HOX *code thus distinguished ASCs from facial and limb depots. Overall, these results strongly suggest that chin and knee fats could have different functional features.

### 3.3. Properties of Chin- and Knee-ASCs and Adipocyte Progenies

**Figure 3:**
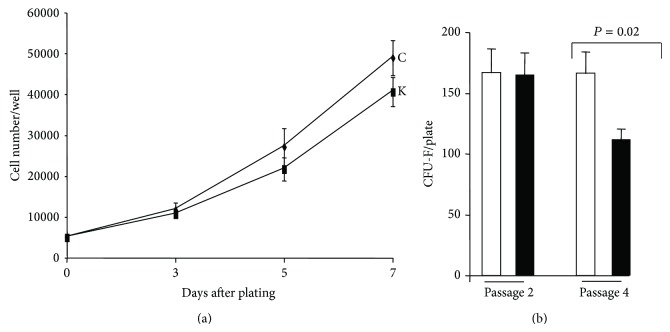
Proliferation and clonogenic potentials of chin and knee ASCs. (a) ASCs isolated from chin (C) and knee (K) were plated at 5000 cells/well in 24-well plates and cell number was quantified at the indicated times. Values are means ± SEM. (*n* = 8 paired depots). (b) Chin-ASCs (open bars) and knee-ASCs (black bars) were plated at a clonal cell density and maintained in proliferative medium. The numbers of colonies were assessed after 10 days of culture. Values are means ± SEM. *P* is indicated. Experiments were performed at cell passage 2 (*n* = 7 paired depots) and at passage 4 (*n* = 6 paired depots).

ASCs and their progenies were characterized in order to determine if there are functional differences between chin and knee fats. We hence compared ASC proliferation, fibroblastoid colony-forming units (CFU-F), and differentiation. Cells were plated at low cell density and allowed to proliferate for 7 days. As shown in Figure 3, the proliferation curve of both ASCs was similar. In contrast, a significant difference in CFU-F potential was observed after cell expansion. Indeed, at passage 2, the CFU-F frequency was identical for both ASCs (16%, which is in the same range as previously reported for other human fat depots [3]). However, after 2 more passages, the CFU-F potential decreased for knee ASCs, whereas it remained unchanged for chin ASCs (Figure 3(b)). These results indicated that both fat depots contained ASCs that could be expanded but that chin ASCs had a higher self-renewal potential, at least* ex vivo.*

**Figure 4:**
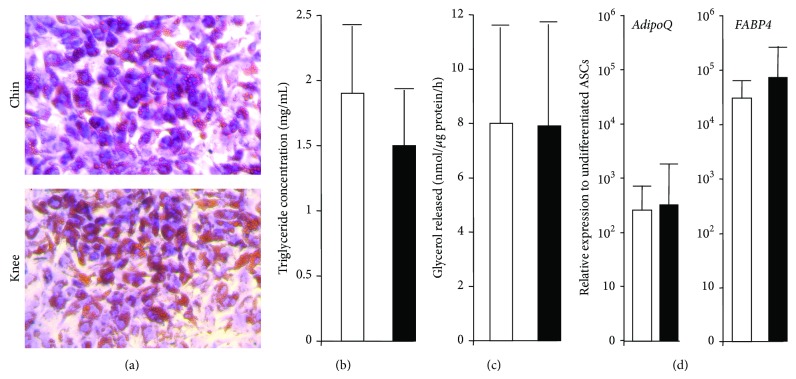
Adipocyte differentiation of chin- and knee-ASCs. ASCs were induced to undergo differentiation for 10 days. (a) Cells were stained with Oil Red O for lipid droplets. (b) Cells were harvested and analyzed for their triglyceride content. (c) Cells were stimulated with forskolin for 4 h and then glycerol release was determined. (d) RNAs were prepared and differentiation levels were assessed by the expression of the indicated genes. Expression of each gene was normalized to* TBP* and then relative to the expression in undifferentiated cells. Values are means ± SEM. *P* is indicated (*n* = 7 paired depots). Open bars: chin; black bars: knee.

**Figure 5:**
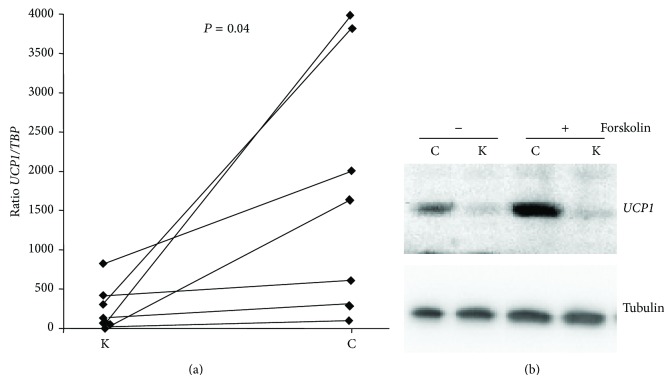
UCP1 expression in adipocytes generated from chin ASCs. ASCs derived from chin (C) and knee (K) were induced to undergo differentiation for 10 days. (a) RNAs were prepared and UCP1 expression was determined by real-time PCR. *n* = 7 paired depots. Corresponding data points of knee and chin paired depots are connected with lines. (b) Cells were stimulated (+) or not (−) with forskolin for 4 h and then proteins were prepared. Expression of UCP1 was assessed by Western-blotting. Tubulin was used as loading control.

Adipocytes generated from ASCs of both depots were then characterized. Adipocytes of both sites accumulated intracellular lipid droplets to a similar extent after 10 days of differentiation, as qualitatively determined by Oil red O staining (Figure 4(a)). Quantitative tests then revealed that adipocytes derived from chin and knee ASCs displayed a similar intracellular triglyceride concentration and lipolysis activity (Figures 4(b) and 4(c)). Expression of adipocyte-specific genes, such as* Adiponectin* (*AdipoQ*) and* FABP4,* was similar for adipocytes generated from both ASCs, supporting at the molecular level that chin- and knee-ASCs have a similar adipogenic capacity (Figure 4(d)). However, a major difference appeared regarding the adipocyte phenotype. As shown in Figure 5(a), the brown adipocyte marker* UCP1 *was highly enriched in chin adipocytes compared to paired knee adipocytes. UCP1 differential expression was confirmed at the protein level (Figure 5(b)). Interestingly, UCP1 expression increased in chin adipocytes upon PKA pathway activation via forskolin treatment (Figure 5(b)), as expected for functional brown adipocytes. Overall, our data indicated that chin host site contained brown or brown-like adipocytes, whereas the knee donor site contained white adipocytes. The presence of functional brown and brown-like adipocytes in human neck has been previously reported [24], and we have observed* UCP1* gene expression in human cheek (not shown). The human face thus contains cells having the potential to enable the dissipation of excessive caloric intake. The tissue regeneration and facial rejuvenation consequences of transplanting white adipocytes in a fat environment with different metabolic properties remain to be investigated.

## 4. Conclusions

Our results strongly suggest that ASCs present in knee and chin fats derived from different embryonic origins. They did not express the same* HOX *code and displayed different adipocyte phenotypes. The consequences of these differences on autologous fat grafting outcomes remain to be investigated in detail. It should be noted that mesenchymal stem cells from different embryonic origins have been found to display different tissue regeneration capacities [25] and that* Hox *code matching was confirmed to be critical for successful tissue regeneration in mouse [14]. Our data highlighted that donor site selection might be an important factor to be considered for fat grafting. Matching* Pax3* and/or* HOX *code and/or* UCP1* expression between host and donor sites could improve postoperative outcomes.

## Figures and Tables

**Table 1:** TOP50 of the down- and upregulated genes between knee and chin adipose tissue depots from the large scale transcriptomic experiment.

Gene symbol	Gene title	Knee versus chin FDR	Fold change
ACTA1	Actin, alpha 1, skeletal muscle	0,0006	−69,62
MYH2	Myosin, heavy chain 2, skeletal muscle, adult	0,0004	−65,45
KBTBD10	Kelch repeat and BTB (POZ) domain containing 10	0,0016	−62,65
MYL1	Myosin, light chain 1, alkali; skeletal, fast	0,0026	−31,4
CKM	Creatine kinase, muscle	0,0026	−29,47
MYBPC1	Myosin binding protein C, slow type	0,0027	−28,89
MB	Myoglobin	0,0025	−25,84
MYL2	Myosin, light chain 2, regulatory, cardiac, slow	0,0029	−24,41
SLN	Sarcolipin	0,0052	−22,39
CSRP3	Cysteine and glycine-rich protein 3 (cardiac LIM protein)	0,0034	−18,46
NEB	Nebulin	0,0037	−17,84
MYOT	Myotilin	0,0046	−16,77
TTN	Titin	0,0069	−16,52
SMPX	Small muscle protein, X-linked	0,0094	−14,54
COL6A6	Collagen, type VI, alpha 6	0,0004	−14,47
KBTBD10	Kelch repeat and BTB (POZ) domain containing 10	0,0093	−14,24
TNNC2	Troponin C type 2 (fast)	0,0043	−13,86
TNNI2	Troponin I type 2 (skeletal, fast)	0,0076	−13,15
TNNC1	Troponin C type 1 (slow)	0,0053	−12,35
TNNT1	Troponin T type 1 (skeletal, slow)	0,0051	−12,08
XIRP2	Xin actin-binding repeat containing 2	0,0086	−11,91
TPM1	Tropomyosin 1 (alpha)	0,0092	−11,73
ENO3	Enolase 3 (beta, muscle)	0,0059	−11,71
PDLIM5	PDZ and LIM domain 5	0,0085	−10,49
ASB5	Ankyrin repeat and SOCS box-containing 5	0,0142	−10,38
MYLPF	Myosin light chain, phosphorylatable, fast skeletal muscle	0,0092	−9,51
CADM2	Cell adhesion molecule 2	5,51*E* − 05	−9,22
MYH6	Myosin, heavy chain 6, cardiac muscle, alpha///myosin	0,0105	−8,65
CA3	Carbonic anhydrase III, muscle specific	0,0006	−8,27
MYBPC2	Myosin binding protein C, fast type	0,0097	−8,06
MYOZ1	Myozenin 1	0,012	−7,9
ACTN2	Actinin, alpha 2	0,0155	−7,86
TRDN	Triadin	0,0075	−7,71
CADM2	Cell adhesion molecule 2	0,0003	−6,45
CYR61	Cysteine-rich, angiogenic inducer, 61	0,0002	−6,29
PAX3	Paired box 3	0,0001	−6,26
TTN	Titin	0,0317	−6,11
CADM2	Cell adhesion molecule 2	0,0003	−5,98
AMPD1	Adenosine monophosphate deaminase 1	0,0243	−5,97
LDB3	LIM domain binding 3	0,0143	−5,96
LMOD2	Leiomodin 2 (cardiac)	0,0218	−5,94
TPM3	Tropomyosin 3	0,0139	−5,9
APOBEC2	Apolipoprotein B mRNA editing enzyme	0,0126	−5,8
C8orf22	Chromosome 8 open reading frame 22	0,008	−5,79
RBM24	RNA binding motif protein 24	0,0006	−5,74
TTN	Titin	0,0171	−5,62
PDLIM5	PDZ and LIM domain 5	0,0325	−5,57
CYR61	Cysteine-rich, angiogenic inducer, 61	0,0002	−5,45
CMYA5	Cardiomyopathy associated 5	0,0152	−5,31
PKIA	Protein kinase (cAMP-dependent, catalytic) inhibitor alpha	0,0038	−5,31
PCDH7	Protocadherin 7	0,0008	−5,23
HSPB3	Heat shock 27kDa protein 3	0,0235	−5,19
PPBP	Proplatelet basic protein (chemokine (C-X-C motif) ligand 7)	0,0153	−5,14
MYH1	Myosin, heavy chain 1, skeletal muscle, adult	0,0276	−5,11
NRAP	Nebulin-related anchoring protein	0,0129	−5,08
POPDC3	Popeye domain containing 3	0,0033	−5,03
MYOZ2	Myozenin 2	0,0209	−4,99
HOXA10	Homeobox A10	1,40*E* − 09	37,88
HOXA9	Homeobox A9	9,16*E* − 08	23,43
HOXA9	Homeobox A9	8,09*E* − 07	13,38
HOXC6	Homeobox C6	1,93*E* − 07	9,92
TNC	Tenascin C	0,0005	8,26
HOXC10	Homeobox C10	3,47*E* − 06	7,8
IGH@///IGHG1///	Immunoglobulin heavy locus	0,0021	7,59
IGJ	Immunoglobulin J polypeptide	0,0083	7,41
HOXA10	Homeobox A10	3,47*E* − 06	7,37
CHI3L1	Chitinase 3-like 1 (cartilage glycoprotein-39)	0,0008	7,09
SAA1///SAA2	Serum amyloid A1///serum amyloid A2	0,0004	7,04
HOXA3	Homeobox A3	5,60*E* − 05	6,88
HOXC9	Homeobox C9	1,32*E* − 06	5,87
CYAT1///IGLV1-44	Cyclosporin A transporter 1/	0,0076	5,83
ASPN	Asporin	1,32*E* − 06	5,76
HOXA5	Homeobox A5	9,27*E* − 05	5,28
ASPN	Asporin	4,94*E* − 06	5,2
CHI3L1	Chitinase 3-like 1 (cartilage glycoprotein-39)	0,0021	4,82
SAA1///SAA2	Serum amyloid A1///serum amyloid A2	0,0011	4,75
ATP8B4	ATPase, class I, type 8B, member 4	2,99*E* − 06	4,61
IGL@	Immunoglobulin lambda locus	0,0058	4,6
CSN1S1	Casein alpha s1	0,0143	4,04
CYAT1///IGLV1-44	Cyclosporin A transporter 1	0,008	3,96
PRG4	Proteoglycan 4	0,001	3,95
IGH@///IGHA1	Immunoglobulin heavy locus	0,0172	3,75
HOXA7	Homeobox A7	5,44*E* − 05	3,69
HOXB7	Homeobox B7	2,43*E* − 05	3,63
IGLV1-44	Immunoglobulin lambda variable 1-44	0,013	3,6
ALDH1A3	Aldehyde dehydrogenase 1 family, member A3	0,0002	3,54
FGFBP2	Fibroblast growth factor binding protein 2	0,0013	3,54
ZIC1	Zic family member 1 (odd-paired homolog, *Drosophila*)	0,0054	3,5
CP	Ceruloplasmin (ferroxidase)	0,0003	3,43
IGK@///IGKC	Immunoglobulin kappa locus t	0,0132	3,39
IGK@///IGKC	Immunoglobulin kappa locus///immunoglobulin kappa constant	0,012	3,37
ISM1	Isthmin 1 homolog (zebrafish)	0,0006	3,36
PHLDA2	Pleckstrin homology-like domain, family A, member 2	0,0011	3,34
NPY1R	Neuropeptide Y receptor Y1	0,0017	3,31
EVI2A	Ecotropic viral integration site 2A	0,0014	3,29
HOXB7	Homeobox B7	2,86*E* − 05	3,29
FCER1A	Fc fragment of IgE, high affinity I, receptor for alpha polypeptide	0,0012	3,28
MSR1	Macrophage scavenger receptor 1	0,0002	3,23
CNTN4	Contactin 4	0,0021	3,23
IGKC	Immunoglobulin kappa constant	0,01	3,21
PLA2G7	Phospholipase A2, group VII (platelet-activating factor acetylhydrolase, plasma)	0,0076	3,21
MS4A7	Membrane-spanning 4 domains, subfamily A, member 7	0,0004	3,19
RNASE6	Ribonuclease, RNase A family, k6	0,0003	3,18
IGK@///IGKC	Immunoglobulin kappa locus///immunoglobulin kappa constant	0,0184	3,18
CRISPLD1	Cysteine-rich secretory protein LCCL domain containing 1	0,0009	3,17
CP	Ceruloplasmin (ferroxidase)	0,0004	3,17
CHI3L2	Chitinase 3-like 2	0,0033	3,12
CLEC7A	C-type lectin domain family 7, member A	0,0205	3,1
LYZ	Lysozyme	0,0123	3,07
BGN	Biglycan	0,0005	3,06
LYZ	Lysozyme	0,0083	2,94
MARCH1	Membrane-associated ring finger (C3HC4) 1	0,0002	2,92
IGK@///IGKC///LOC100291464	Immunoglobulin kappa locus///immunoglobulin kappa constant///similar to hCG26659	0,0172	2,91
SAMSN1	SAM domain, SH3 domain, and nuclear localization signals 1	0,0013	2,91
ANKDD1A	Ankyrin repeat and death domain containing 1A	0,0006	2,88

## References

[1] Coleman S. R. (1995). Long-term survival of fat transplants: controlled demonstrations. *Aesthetic Plastic Surgery*.

[2] Kølle S.-F. T., Fischer-Nielsen A., Mathiasen A. B. (2013). Enrichment of autologous fat grafts with ex-vivo expanded adipose tissue-derived stem cells for graft survival: a randomised placebo-controlled trial. *The Lancet*.

[3] Bourin P., Bunnell B. A., Casteilla L. (2013). Stromal cells from the adipose tissue-derived stromal vascular fraction and culture expanded adipose tissue-derived stromal/stem cells: a joint statement of the International Federation for Adipose Therapeutics and Science (IFATS) and the International Society for Cellular Therapy (ISCT). *Cytotherapy*.

[4] Yoshimura K., Sato K., Aoi N. (2008). Cell-assisted lipotransfer for facial lipoatrophy: efficacy of clinical use of adipose-derived stem cells. *Dermatologic Surgery*.

[5] Fredriksson J. M., Thonberg H., Ohlson K. B. E., Ohba K.-I., Cannon B., Nedergaard J. (2001). Analysis of inhibition by H89 of UCP1 gene expression and thermogenesis indicates protein kinase A mediation of *β*3-adrenergic signalling rather than *β*3-adrenoceptor antagonism by H89. *Biochimica et Biophysica Acta—Molecular Cell Research*.

[6] Enerbäck S. (2010). Human brown adipose tissue. *Cell Metabolism*.

[7] Tchkonia T., Giorgadze N., Pirtskhalava T. (2002). Fat depot origin affects adipogenesis in primary cultured and cloned human preadipocytes. *The American Journal of Physiology—Regulatory Integrative and Comparative Physiology*.

[8] Tchkonia T., Tchoukalova Y. D., Giorgadze N. (2005). Abundance of two human preadipocyte subtypes with distinct capacities for replication, adipogenesis, and apoptosis varies among fat depots. *American Journal of Physiology—Endocrinology and Metabolism*.

[9] Billon N., Dani C. (2012). Developmental origins of the adipocyte lineage: new insights from genetics and genomics studies. *Stem Cell Reviews and Reports*.

[10] Gesta S., Blüher M., Yamamoto Y. (2006). Evidence for a role of developmental genes in the origin of obesity and body fat distribution. *Proceedings of the National Academy of Sciences of the United States of America*.

[11] Billon N., Iannarelli P., Monteiro M. C. (2007). The generation of adipocytes by the neural crest. *Development*.

[12] Krumlauf R. (1994). *Hox* genes in vertebrate development. *Cell*.

[13] Wang K. C., Helms J. A., Chang H. Y. (2009). Regeneration, repair and remembering identity: the three Rs of Hox gene expression. *Trends in Cell Biology*.

[14] Leucht P., Kim J.-B., Amasha R., James A. W., Girod S., Helms J. A. (2008). Embryonic origin and Hox status determine progenitor cell fate during adult bone regeneration. *Development*.

[15] Creuzet S., Couly G., Vincent C., le Douarin N. M. (2002). Negative effect of Hox gene expression on the development of the neural crest-derived facial skeleton. *Development*.

[16] White P., Thomas D. W., Fong S. (2003). Deletion of the homeobox gene PRX-2 affects fetal but not adult fibroblast wound healing responses. *Journal of Investigative Dermatology*.

[17] Bolstad B. M., Irizarry R. A., Åstrand M., Speed T. P. (2003). A comparison of normalization methods for high density oligonucleotide array data based on variance and bias. *Bioinformatics*.

[18] Rodriguez A.-M., Pisani D., Dechesne C. A. (2005). Transplantation of a multipotent cell population from human adipose tissue induces dystrophin expression in the immunocompetent mdx mouse. *The Journal of Experimental Medicine*.

[19] Wdziekonski B., Mohsen-Kanson T., Villageois P., Dani C. (2011). The generation and the manipulation of human multipotent adipose-derived stem cells. *Methods in Molecular Biology*.

[20] Benjamini Y., Hochberg Y. (1995). Controlling the false discovery rate: a practical and powerful approach to multiple testing. *Journal of the Royal Statistical Society Series B: Methodological*.

[21] Betters E., Liu Y., Kjaeldgaard A., Sundström E., García-Castro M. I. (2010). Analysis of early human neural crest development. *Developmental Biology*.

[22] Tchkonia T., Lenburg M., Thomou T. (2007). Identification of depot-specific human fat cell progenitors through distinct expression profiles and developmental gene patterns. *American Journal of Physiology—Endocrinology and Metabolism*.

[23] Cantile M., Procino A., D'Armiento M., Cindolo L., Cillo C. (2003). HOX gene network is involved in the transcriptional regulation of in vivo human adipogenesis. *Journal of Cellular Physiology*.

[24] Cypess A. M., White A. P., Vernochet C. (2013). Anatomical localization, gene expression profiling and functional characterization of adult human neck brown fat. *Nature Medicine*.

[25] Uccelli A., Moretta L., Pistoia V. (2008). Mesenchymal stem cells in health and disease. *Nature Reviews Immunology*.

